# Investigation of the Effects of Microbiota on Exercise Physiological Adaption, Performance, and Energy Utilization Using a Gnotobiotic Animal Model

**DOI:** 10.3389/fmicb.2019.01906

**Published:** 2019-08-20

**Authors:** Wen-Ching Huang, Yi-Hsun Chen, Hsiao-Li Chuang, Chien-Chao Chiu, Chi-Chang Huang

**Affiliations:** ^1^Department of Exercise and Health Science, National Taipei University of Nursing and Health Sciences, Taipei, Taiwan; ^2^Graduate Institute of Veterinary Pathobiology, National Chung Hsing University, Taichung, Taiwan; ^3^National Laboratory Animal Center, National Applied Research Laboratories, Taipei, Taiwan; ^4^Graduate Institute of Sports Science, National Taiwan Sport University, Taoyuan, Taiwan

**Keywords:** gnotobiote, butyrogenic, exercise, energy availability, physiological adaption

## Abstract

The wide diversity in gut microbiota that is found among individuals is affected by factors including environment, genetics, dietary habits, and lifestyle after birth. The gastrointestinal tract, the largest and most complicated *in vivo* ecosystem, is a natural habitat for microbe colonization. Gut microbiota acts as “metabolic organ” that interacts with the human host symbiotically and performs an important role in maintaining health. In addition to the above factors, microbiota distributions/proportions are affected by exercise and other forms of physical activity. However, diet, lifestyle, and nutritional supplementation may impede the actual analytic relationship in practice. Therefore, the purpose of this study is to understand the effects of several microbiota on physical fitness, exercise performance, energy metabolism, and biochemistries using the concept of gnotobiote based on a germ-free model. The microbes *Eubacterium rectale, Lactobacillus plantarum* TWK10*, and Clostridium coccoides* were separately inoculated into gnotobiotic animal models. Fecal analysis was regularly done for the entire duration of the experiment. The exercise capacities were measured repeatedly with and without aerobic exercise training using an exhaustive swimming test. Various fatigue-associated biochemical variables, including lactate, ammonia, glucose, lactic dehydrogenase (LDH), and creatine kinase (CK) were also measured to assess physiological adaption. In addition, metabolic phenotype was applied to record basal metabolic rate, diet, behavior, and activities. Body composition, glycogen content, and histopathology were further evaluated to assess the gnotobiotic effects. *E. rectale* engendered capacities, physiological adaption, and physical activities that were significantly better than other two microbes, possible due to energy regulation and bioavailability. In addition, *L. plantarum* TWK10 and *C. coccoides* were found to significantly increase the basal metabolic rate and to alter the body compositions, although no exercise capacity benefit was found in the gnotobiotic models. The *E. rectale* and *L. plantarum* gnotobiotic animals all showed normal histological observations with the exception of the *C. coccoides* gnotobiote, which showed the pathological observation of hepatic necrosis. The gnotobiotic model directly demonstrates the interactions between microbes and hosts, which are especially relevant and applicable to the field of sports science. This study supports the development of beneficial microbiota for application to exercise and fitness, which is an emerging area of health promotion.

## Introduction

Gut microbiota develops its specific diversity in individuals based on factors that include environment, genetics, dietary habits, and lifestyle after birth (Schippa and Conte, 2014). The gastrointestinal tract (GI tract), a natural habitat for 10–100 trillion microbes, is the largest and most complicated *in vivo* ecosystem. Moreover, the GI contains the most completed neurosystem connected to brain, forming the gut-brain axis. The microbiota has been shown to affect a wide range of mammalian neurotransmitters, including dopamine, norepinephrine, serotonin, and gamma-aminobutyric acid (GABA) (Strandwitz, 2018), which impact host physiology and neuro-related diseases (Dinan and Cryan, 2017). Therefore, the gut microbiota eubiosis influences the well-being of the host by contributing to its metabolism, physiology, nutrition, and immune functions. The intervention of probiotics/prebiotics is an emerging therapeutic strategy targeting dysbiosis-associated diseases (Vemuri et al., 2017; Kriss et al., 2018).

Previously, specific exercise prescriptions have elucidated health-promotion and disease prevention/amelioration benefits, possibly supporting the biological basis for a gut-muscle axis (Grosicki et al., 2018). In addition, Mach et al. proposed that microbiota plays important roles in endurance, exercise-associated oxidative stress, inflammation, and energy balance during intense exercise (Mach and Fuster-Botella, 2017). Furthermore, exercise/physical activity may modulate the microbiota succession via a variety of factors, including bile acid, short-chain fatty acids (SCFAs), immunoglobin, myokine, weight control, and hormone change (Cerdá et al., 2016). One report has suggested that both diet and exercise affect microbiota compositions via interactions between the gut-brain and gut-muscle axis (O’Sullivan et al., 2015), while another elucidated the strong influence of exercise on gut integrity, finding that the host microbiome potentially influences *Faecalibacterium prausnitzi*, *Clostridium* spp., and *Allobaculum* spp. specific bacteria between the gut and the host (Campbell et al., 2016). Moreover, another study found a significantly raised *Firmicute*, *Lactobacillaceae*, and *Lactobacillus* composition in elite athletes who do regular exercise training compared to a population of healthy individuals (Clarke et al., 2014). Therefore, exercise may increase microbiota diversity independent of diet, with the capacity potentially influenced by the presence of a diverse microbiota (Campbell and Wisniewski, 2017).

The different population, elderly and adult, exhibited the decrease in the diversity of the microbiota, characterized by a large interindividual variability, with lower numbers of *Firmicutes*, *Bifidobacteria*, *Clostridium* cluster XIV, *Faecalibacterium Prausnitzii*, *Blautia coccoides* (*C. coccoides*)–*Eubacterium rectale* and higher presence of Enterobacteriaceae and Bacteroidetes (Rondanelli et al., 2015). The exercise intervention could also modulate the serum leptin and ghrelin levels by increasing in the number of *Lactobacillus*, *Bifidobacterium* and *B. coccoides*–*E. rectale* group (Queipo-Ortuño et al., 2013). Besides, the previous study also showed the *Lactobacillus plantarum* TWK10 supplement could elevate the exercise capacities possibly by energy regulation and muscular development in mice models (Chen et al., 2016). The short-chain fatty acids (SCFAs), produced by butyrogenic microbe, could be important energy substrate during endurance exercise (Okamoto et al., 2019) and *E. rectale* was also validated its butyrogenic characteristics by different prebiotic stimulations (Scott et al., 2014).

Germ-free (GF) animals provide an important and unique experimental platform to directly investigate the interactions between a host and its microbiota. The term “germ-free” refers to an animal demonstrably free from microbes, including bacteria, viruses, fungi, protozoa, and parasites, throughout its lifetime and maintained in a specific environment. Thus, GF animal models have been widely applied to study host–microbiota interactions in fields such as cancer biology, neurobiology, behavior, neurogastroenterology, cardiology, reproductive biology, energy metabolism, and bone homeostasis (Al-Asmakh and Zadjali, 2015). Gnotobiotic animals, which are established by colonizing specific/known microbes in GF mice, are used to reveal causal relationships between known microbes and a specific topic of interest. The microbes, including *L plantarum, C. coccoides* and *E. rectale*, demonstrated the significant succession after indicated conditions, especially in exercise, health status, and population differences. Therefore, we could apply the current gnotobiotic animal model to directly investigate the relationship of indicated microbes and functional effects.

This study, guided by previous reports and our recent study (Tan et al., 2014; Campbell et al., 2016; Chen et al., 2016, 2017), was designed to investigate the effects of microbes on exercise physiology using a gnotobiotic animal model. Few studies in the literature have examined the effects of individual/single microbes on exercise performance, training physiological adaption, energy expenditure, behaviors, and pathology. Thus, this study hoped to use this platform to identify potential probiotics and/or microbes and then determine their direct physiological effects from perspective of sport science and microbiota.

## Materials and Methods

### Materials

*Eubacterium rectale* (ATCC^®^, 33656) and *Clostridium coccoides (also called Blautia coccoides*, ATCC^®^, 29236) microbes were purchased from Biosource Collection and Research Center (BCRC, Hsinchu, Taiwan) and *Lactobacillus plantarum* TWK10 microbes were purchased from Synbio Tech Inc. (Kaohsiung, Taiwan). The *E. rectale* and *C. coccoides* were cultured with Tryoticase soy agar (TSA) in the anaerobic incubator at 37°C for 3 days, respectively, then collected colonies and dilute to the concentration required for the study. The *L. plantarum* TWK10 was cultured with MRS agar in the incubator at 37°C for 2 days. These microbes were prepared for gnotobiotic model establishment.

### Animals and Experimental Designs

Male C57BL/6JNarl mice (6 weeks old, germ-free level) purchased from National Laboratory Animal Center (Taipei, Taiwan) were used in this study. These animals were verified and maintained in a vinyl isolator to insure their germ-free status. The mice (*n* = 6–8/each microbe) were assigned to individual isolators and left undisturbed for a period of 1 week for dietary and environment acclimation. The animals were fed a sterile diet (#5010, PMI Nutrition International, St. Louis, MO, United States) and water *ad libitum* during the experiments. Environmental conditions maintained a steady photoperiod, humidity, and temperature (12-h light/12-h dark cycle, 55–65%, and 24 ± 2°C, respectively). This study was carried out in accordance with the principles of the Basel Declaration and the recommendations of the Institutional Animal Care and Use Committee (IACUC) of National Taiwan Sport University. The protocol (IACUC-10612) was approved by the IACUC of National Taiwan Sport University.

**FIGURE 1:**
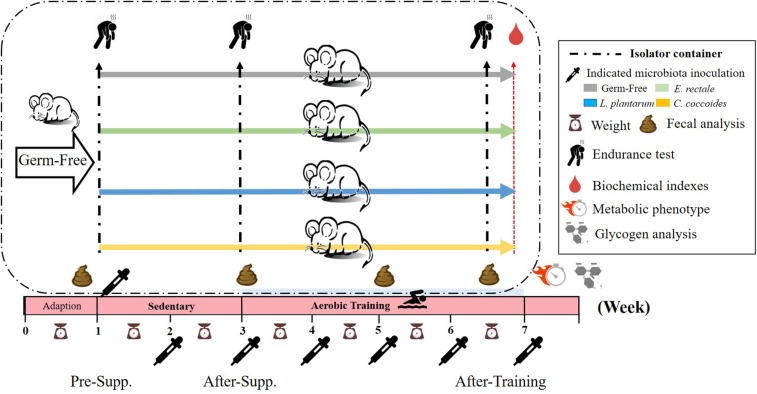
Experimental designs to assess the exercise-capacity and physiological-adaption effects on gnotobiotic animals. The GF animals were randomly assigned, inoculated, and colonized with the indicated 4 groups (GF, *E. rectale*, *L. plantarum* TWK10, and *C. coccoides*). Fecal samples were regularly analyzed and the physical capacities and related biochemistries were assessed within the test duration. The metabolic phenotype, body compositions, and glycogen were measured at the end of study. Supp., supplementation.

The experimental protocol is presented in Figure 1. After the 1-week acclimation period, the germ-free status of the mice was verified by fecal examination. Next, the mice were inoculated with *E. rectale*, *C. coccoides*, and *L. plantarum* TWK10 at about 10^10 CFU/0.5 mL each by oral gavage every weeks. Gut bacterial composition was monitored for gnotobiotic animal characteristics. The exercise capacity of the gnotobiotic animals were assessed in the absence of aerobic exercise training. Subsequently, the gnotobiotic mice were subjected to a programed aerobic training based on a regimen that was slightly modified from our previous study (Chen et al., 2014). Exercise capacity was evaluated using aerobic endurance capacities and the exercise-related biochemistry was immediately assessed after a fixed exercise intensity for exercise physiological adaption.

### Gut Bacterial Composition

Mice were housed in sterilized flexible film isolators system (1.5 m × 0.6 m × 0.6 m) (CBC, Madison, WI, United States) and the environment was routinely evaluated by fecal samples, bedding and drinking water by standard microbiological techniques to detect any contamination by bacteria, viruses, molds, and yeasts for the germ-free or gnotobiotic status (Chuang et al., 2012). The fresh fecal samples from four individual groups were collected at 1, 3, 5, and 7 weeks, respectively, before and after oral inoculation. Each fresh fecal sample was first normalized to 20 mg/mL under anaerobic conditions, 10-fold dilution was performed under anaerobic operation box (Forma Anaerobic system, Thermo Scientific), and 0.05-mL samples were inoculated onto each of 5 non-selective and 13 selective agar media. Isolated bacteria were identified by analyzing colony and cell morphology, aerobic growth, and spore formation as well as by Gram staining (Itoh and Mitsuoka, 1980; Itoh et al., 1983; Yanabe et al., 2001).

### Aerobic Endurance Training and Performance Test

Endurance performance using survival motives was used to assess aerobic capacities. In a separated isolator, the mice were forced to swim in a tank until exhaustion without weight loading. The persistent time (number of seconds from start to exhaustion) was recorded and used as the aerobic endurance performance index. The details of the procedures and protocol were described in our previous article with modifications (Huang et al., 2018b). For the aerobic endurance training, all the mice (Four groups) were subjected to swimming training 10 min/day and 5 days/week with necessary assistance (week 3–7) in the indicated isolator.

### Determination of Fatigue-Associated Biochemical Indexes

The fatigue-associated variables were measured using exercise intensity interventions after fasting for 8 h in order to reflect the real physiological adaption under exercise. For the indexes for lactate, ammonia, glucose, CK, and LDH, the blood was sampled immediately after 5 min of acute exercise. The methods were modified for current animal characteristics based on our previous study (Ho et al., 2017). The blood samples that had fully coagulated were centrifuged at 1000 × *g* and 4°C for 15 min for serum separation and assessed using an autoanalyzer (Hitachi 7060, Hitachi, Tokyo, Japan).

### Metabolic Phenotype Characteristics

At the end of the experiment, animals were housed individually in metabolic phenotyping chambers (Mouse Promethion Continuous Caging System; Sable Systems^TM^, Las Vegas, NV, United States) and maintained on a standard 12 h light/dark cycle. In addition, food and water were provided *ad libitum*, with amounts recorded continuously. Variables, including energy expenditure, food and water uptake, meal and drinking patterns, total activity and wheel-running, and live body mass, were also monitored for 3 days in order to elucidate the effects of the introduced microbes on the gnotobiotic animal model.

### Body Composition and Glycogen Content Analysis

The indicated microbes were administrated for 6 weeks until animal sacrifice. All of the mice were euthanatized by 95% CO2 asphyxiation. After sacrifice, the important visceral organs, including liver, kidney, heart, lung, muscle, spleen, cecum, and EPF, were accurately excised and weighed. Then, the organs were saved in 10% formalin for further histopathology. A portion of muscle and liver tissues was stored in liquid nitrogen for later glycogen content analysis and real-time PCR for specific gene expression. The 100 mg of liver and muscle was finely cut, weighed, and homogenized in 0.5 ml cold perchloric acid for further glycogen analysis. The homogenate was centrifuged for 15 min at 15000 × *g* at 4°C. The supernatant was carefully decanted and kept on ice. A standard glycogen or tissue extract, 30 μL, was added to 96-well microplates, and iodine-potassium iodide reagent, 200 μL, was added to each well for binding iodine to glycogen. An amber-brown compound developed immediately after the reaction. Absorbance was measured at wavelength 460nm with use of an ELISA reader after the material rested for 10 min (Huang et al., 2012).

### Quantitative Real-Time Polymerase Chain Reaction (RT-PCR)

After sacrifice, a portion of the muscle was prepared in order to first extract the total cellular RNA using RNeasy Mini Kit (Qiagen, Waltham, MA, United States) in accordance with the manufacturer’s instructions and then to reverse transcribe the material into complimentary DNA(cDNA) using MMLV reverse transcriptase (Promega, Madison, WI, United States). The expression of glucose transporter 1 (GLUT1), glucose transporter 4 (GLUT4), and β-actin were quantified using qRT-PCR. Respective primer sets (forward and reverse) referred to previous study (Hu et al., 2014) as follows: GLUT4, 5-TGCTCTCCT GCAGCTGATT-3, and 5-TTCAGCTCAGCTAGTGCGTC-3; GLUT1, 5-CTTCCTGCTCATCAATCGT-3, and 5-AGCTCCA AGATGGTGACCTT-3; and Beta-actin, 5-CTAAGGCCAACC GTGAAAAG-3, and 5-ACCAGAGGCATACAGGGACA-3. Levels of GLUT4 and GLUT1 were normalized against the amount of Beta-actin mRNA. Reactions were performed on a LightCycler 1.5 using a LightCycler TaqMan master kit (both from Roche, Mannheim, Germany). Preincubation was performed at 95°C for 10 min, followed by 40 cycles of denaturation at 95°C for 10 s, annealing at 60°C for 30 s, and extension at 72°C for 1 s, and cooling at 40°C for 30 s. LightCycler^®^ Software 3.5 (Roche) was used to perform relative quantification using beta-actin as an internal control.

### Histopathology

The visceral organs that had been preserved in 10% formalin were trimmed in tissue sections of 4 μm thickness slices and embedded in paraffin. These sections were further stained with hematoxylin and eosin (H&E) and examined by a veterinary pathologist under a light microscope equipped with a CCD camera (BX-51, Olympus, Tokyo, Japan).

### Statistical Analysis

The data were represented as mean ± SEM. The statistical differences among the groups in terms of exercise capacity, biochemistry, body composition, gene expression, metabolic phenotype indexes, and glycogen content were analyzed using one-way analysis of variance (ANOVA). In addition, two-way mixed ANOVA (indicated microbe × time) was applied to the exercise capacity difference curve profile and exercise capacity was compared using a within-group paired *t*-test. The pathological score was also statistically evaluated by Kruskal–Wallis test for the difference among groups. The statistical analysis used SPSS v. 19.0 and data were considered statistically significant when the probability of a type I error was less than 0.05.

## Results

### The Effects of Microbiota on Endurance Capacity

**FIGURE 2:**
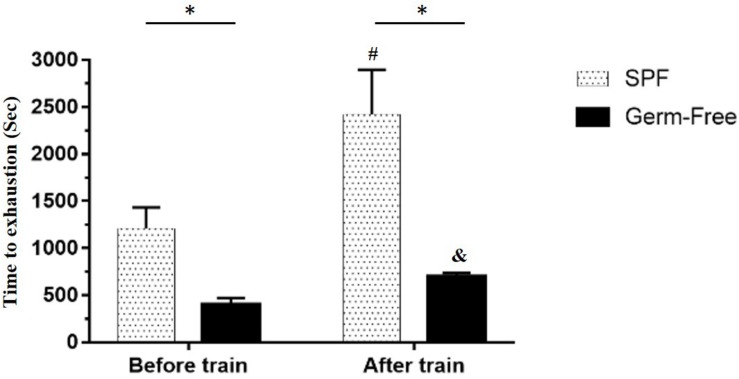
Effect of microbiota on exercise capacity and adaption. Data are represented as mean ± SEM (*n* = 8/group). ^∗^*P* < 0.05, compared between groups. ^#^*P* < 0.05, compared within SPF group. ^&^*P* < 0.05, compared within GF group.

The GF mice scored significantly lower than the untrained SPF mice (*P* < 0.05). Furthermore, a significant difference was observed after aerobic exercise training (*P* < 0.05) using the independent *t*-test. Based on the paired *t*-test, the GF mice after training scored significantly higher than those prior to training within groups. A similar level of significance was found in the SPF mice. After two-way mixed ANOVA analysis, both the training and microbiota main effects demonstrated significant differences (*P* < 0.05), and the interaction effect exhibited a significant difference [*F*(1,28) = 23.8, *P* = 0.038, η = 0.350; Figure 2].

### Colonization by Indicated Microbes and Establishing Gnotobiotic Animals

As shown in Figure 1, the indicated microbes were inoculated at 10^10 CFU by oral gavage after the 1-week acclimation period. Fecal analysis and replenishment were conducted every 2 weeks in order to verify gnotobiotic establishment. Table 1 shows that the indicated gnotobiotic animals were established over the given duration in order to reliably realize the possible gnotobiotic effects in the subsequent experiments. *L. plantarum* and *C. coccoides* did not colonize stably in rodent intestines because the fecal analysis did not show significant increases of these microbes in repeated ANOVA analysis. However, *E. rectale* showed a significant incremental increase over time [*F*(2,10) = 23.85, *P* = 0.018, η = 0.674].

**TABLE 1:** The microbiota colonization in indicated gnotobiotic animals.

**Microbiota**	**Inoculation**	**Fecal (1st)**	**Fecal (2nd)**	**Fecal (3rd)**
Germ Free	saline	0	0	0
*E. rectale*	1 (10^10)	6.8 ± 0.1^a^(10^5)	8.2 ± 0.2^b^(10^5)	1.2 ± 0.2^c^(10^6)
*L. plantarum*	1 (10^10)	7.8 ± 0.3 (10^8)	7.6 ± 0.2 (10^8)	7.8 ± 0.2 (10^8)
*C. coccoides*	1 (10^10)	6.4 ± 0.1 (10^6)	6.5 ± 0.3 (10^6)	6.8 ± 0.6 (10^6)

*The indicated microbiota was inoculated 0.5 mL by oral gavage and simultaneously analyzed the fecal for specific microbiota content every 2 weeks. The unit of table was represented as CFU/g (fecal). Values in the same row with different superscript letters (a,b,c) differ significantly, *p* < 0.05, by repeated ANOVA.*

### The Effects of the Indicated Microbes on Exercise Endurance in the Gnotobiotic Model

**FIGURE 3:**
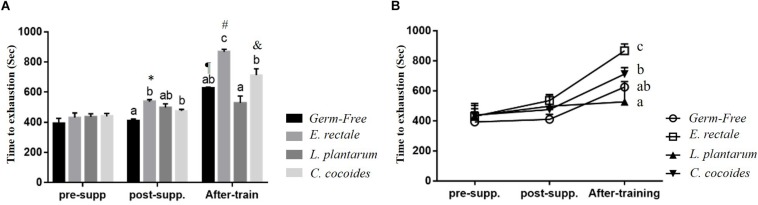
Effect of 6-week indicated microbe supplementation on exercise capacity. Exercise endurance at different time points **(A)** and Exercise endurance profile **(B)**. Data are mean ± SEM (*n* = 6–8/group). Columns with different superscript letters (a,b) are significantly different at *P* < 0.05 at the same time point. ^∗^*P* < 0.05, compared within *E. rectale* group (pre-supp vs. post-supp). ^¶^ *P* < 0.05, ^#^*P* < 0.05, and ^&^*P* < 0.05 compared within GF, *E. rectale*, and *C. coccoides* group (post-supp vs. after-train), respectively.

Initial endurance capacity in the GF mice were assessed after 1 week, with no significant intergroup difference identified [*F*(3,32) = 1.966, *P* = 0.139] at beginning of experiment (Figure 3A). After 2 weeks, a significant intergroup difference [*F*(3,32) = 3.409, *P* = 0.029] was identified, with the *E. rectale* and *C. coccoides* groups significantly higher than the GF group (*P* < 0.05). The *E. rectale* group was also shown to have significantly improved (*P* = 0.043) using a within-group paired *t*-test (pre-supp vs. after-supp). After programed aerobic swimming training, all of the groups except for the *L. plantarum* group (*P* = 0.078) increased significantly in terms of within-group endurance capacity (*P* < 0.05). A significant difference among groups [*F*(3,32) = 13.45, *P* < 0.0001, η = 0.590] was also observed after training. The *E. rectale* group was significantly higher than other three groups (*P* < 0.05), but the *L. plantarum* and *C. coccoides* groups did not differ significantly from the GF group (*P* > 0.05).

Figure 3B illustrates that endurance profiles increased based on the two factors of inoculated microbe and exercise training intervention. The main effects of training and microbes differed significantly [*F*(1,28) = 71.96, *P* < 0.0001, η = 0.72; *F*(3,28) = 14.79, *P* < 0.0001, η = 0.613]. The effect of the *E. rectale* treatment was significantly higher than that in the other groups. In addition, the interaction effect showed a significant difference [*F*(3,28) = 5.78, *P* = 0.003, η = 0.382], which is consistent to the result shown in Figure 1 on the exercise effects in normal microbiota and GF animals.

### Gnotobiotic Animal Exercise-Related Biochemical Indexes, Post-exercise Challenge

**FIGURE 4:**
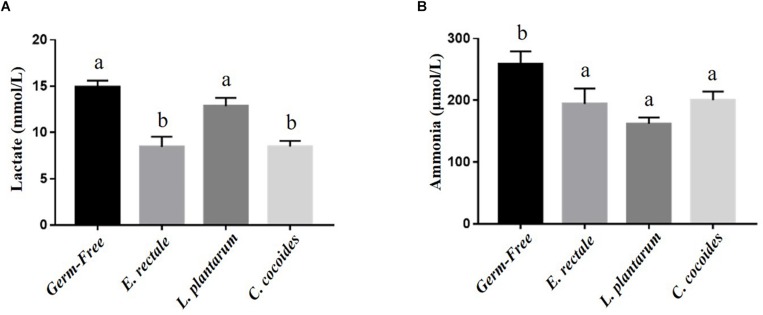
Effect of indicated gnotobiotic groups on the serum lactate **(A)** and ammonia **(B)** levels after the exercise challenge. Data are mean ± SEM (*n* = 6–8/group) and the columns with different superscript letters (a,b) are significantly different at *P* < 0.05.

**FIGURE 5:**
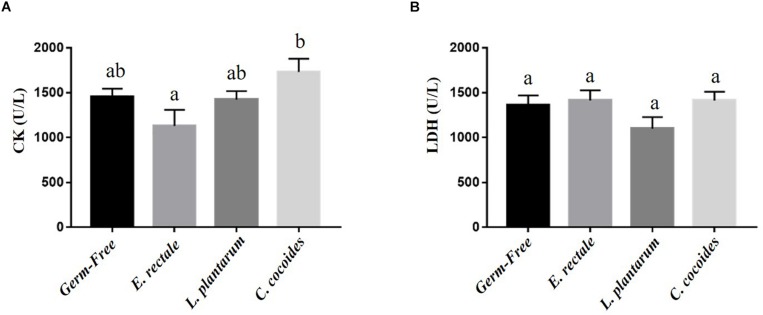
Effect of indicated gnotobiotic groups on the serum CK **(A)** and LDH **(B)** levels after the exercise challenge. Data are mean ± SEM (*n* = 6–8/group) and the columns with different superscript letters (a,b) are significantly different at *P* < 0.05.

Metabolites such as lactate and ammonia were highly associated with physiological status and directly reflected energy utilization, balance, and metabolism during exercise. Significant differences in lactate level were observed among the groups [Figure 4A; *F*(3,32) = 15.37, *P* < 0.0001, η = 0.64], with the *E. rectale* and *C. coccoides* groups significantly higher than the GF and *L. plantarum* groups (*P* < 0.05). In addition, ammonia levels differed significantly among the groups [*F*(3,32) = 4.34, *P* = 0.014, η = 0.343], with the GF groups increasing more significantly than the other three groups (*P* < 0.05), which showed no significant intergroup difference. Exercise-induced injury indexes, including CK and LDH, are often applied to evaluate muscular and tissue integrity during or after exercise. A significant intergroup difference was identified in terms of CK level [*F*(3,32) = 3.446, *P* = 0.031, η = 0.294] but not in terms of LDH level [*F*(3,32) = 1.814, *P* = 0.173, η = 0.191] (Figure 5). In analyzing the CK index differences further, the *E. rectale* group scored significantly less than the *C. coccoides* group, but no significance difference was found between the GF and *L. plantarum* groups.

### Gnotobiotic Animal Energy Utilization Effects, Post-exercise Challenge

**FIGURE 6:**
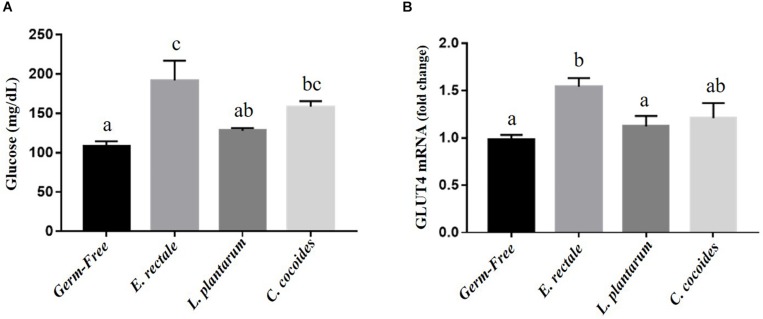
Effect of indicated gnotobiotic groups on the serum glucose **(A)** after the exercise challenge and muscular GLUT4 expression levels **(B)**. Data are mean ± SEM (*n* = 6–8/group) and the columns with different superscript letters (a,b) are significantly different at *P* < 0.05.

Glucose is one the most important fuels for meeting exercise-related energy demands. After the exercise intervention, glucose levels registered significant intergroup differences [*F*(3,32) = 7.59, *P* = 0.001, η = 0.191] (Figure 6A), with the *E. rectale* and *C. coccoides* groups significantly higher than the GF group (*P* < 0.05) and no significant difference found between the GF and *L. plantarum* groups. The expression of muscle tissue in glucose transporters (GLUT1 and GLUT4) was also analyzed to assess energy bioavailability. Significant intergroup differences for GLUT4 [*F*(3,32) = 3.89, *P* = 0.035, η = 0.275] but not for GLUT1 [*F*(3,32) = 1.25, *P* = 0.252, η = 0.345] were found (data not shown), with the *E. rectale* group showing significantly higher GLUT4 values than the GF and *L. plantarum* groups (*P* < 0.05; Figure 6B).

### Gnotobiotic Animal Glycogen Content Effects

**FIGURE 7:**
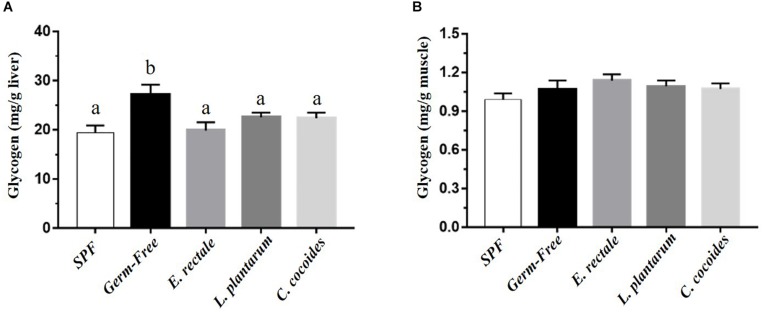
Effect of indicated gnotobiotic groups on hepatic **(A)** and muscle **(B)** glycogen level. Data are mean ± SEM (*n* = 6–8/group) and the columns with different superscript letters (a,b) are significantly different at *P* < 0.05.

Glycogen is synthesized via the glycogenesis pathway for the purpose of energy supply and homeostasis, with the liver and muscle serving as primary storage sites. While glycogen content in the liver is higher than in the muscles in terms of percentage of mass, muscles contain the majority (three-quarters) of total glycogen volume in the body. Gnotobiotic effects were shown to affect glycogen content in the liver [Figure 7A; *F*(4,32) = 4.18, *P* = 0.008] but not in muscles [Figure 7B; *F*(4,32) = 1.10, *P* = 0.373]. Hepatic glycogen in the GF group was significantly higher than in the SPF, *E. rectale*, *L. plantarum*, and *C. coccoides* groups (*P* < 0.05).

### Gnotobiotic Animal Metabolic Phenotype Indexes

The environment in the metabolic phenotype cage was similar to that of the original cages, with sterile bedding and a standard diet provided in order to gather synchronized and consecutive metabolic and behavioral information on the targeted effects in the gnotobiotic animals. A significant intergroup difference in basal metabolic rate (BMR) was found [*F*(3,18) = 21.39, *P* < 0.0001], with the *L. plantarum* and *C. coccoides* groups significantly higher than the other two groups (*P* < 0.05; Table 2). Dietary uptake information was also recorded and used to calculate caloric intake, which was found not to differ significantly among the groups [*F*(3,18) = 1.417, *P* = 0.272]. Physical activity was also monitored using motion sensors and a wheel device. Significant differences among the groups in terms of wheel running distance [*F*(3,18) = 13.89, *P* < 0.0001] and activity proportions [*F*(3,18) = 6.21, *P* = 0.005] were observed. In terms of wheel running distance, the gnotobiotic groups were significant higher than the GF group, and the *E. rectale* group was significantly higher than the *L. plantarum* and *C. coccoides* groups (*P* < 0.05). The proportions of physical activities in the gnotobiotic groups were also significantly higher than the GF group.

**TABLE 2:** The metabolic phenotype indexes in indicated gnotobiotic animals.

**Microbiota**	**BMR (kcal/hr)**	**Diet (g)**	**Distance**	**Activity (%)**
Germ Free	0.463 ± 0.014^a^	2.35 ± 0.11	1011 ± 062^a^	17.3 ± 1.1^a^
*E. rectale*	0.467 ± 0.009^a^	2.79 ± 0.31	2166 ± 158^b^	26.7 ± 1.8^b^
*L. plantarum*	0.531 ± 0.011^b^	2.38 ± 0.08	1579 ± 192^b^	27.0 ± 3.4^b^
*C. coccoides*	0.522 ± 0.007^b^	2.40 ± 0.10	1527 ± 086^b^	26.2 ± 0.8^b^

*Values in the same column with different superscript letters (a,b) differ significantly, *p* < 0.05, by one-way ANOVA. BMR, basal metabolic rate; Distance, wheel running (m/12 h).*

### Gnotobiotic Animal Growth Curve and Body Composition Effects

**FIGURE 8:**
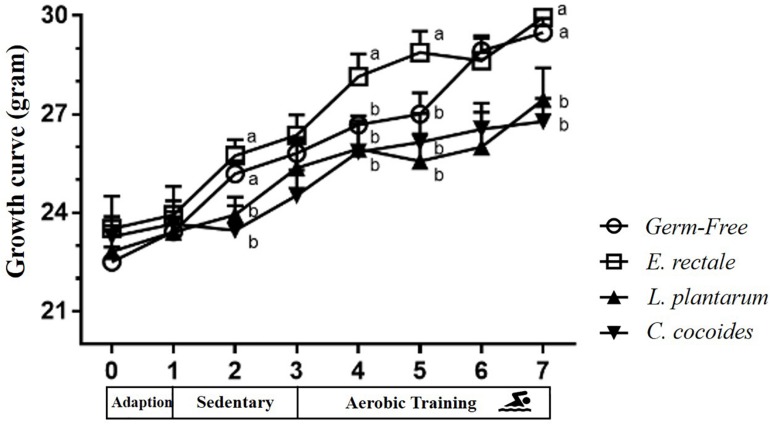
Effect of indicated gnotobiotic groups on growth curve profiles. Data are mean ± SEM (*n* = 6–8/group) and the symbols with different superscript letters (a, b, and c) are significantly different at *P* < 0.05 at the same time.

The toxicity of administration may be assessed in accordance with OECD Guideline 407 using several parameters, including behavior, diet, growth curve, organ weight, and histopathology. Social behavior was normal based on daily observation by a veterinarian and physical activity differed significantly based on quantitative monitoring (Table 2). The growth curve (Figure 8) demonstrated that the indicated microbe supplementation and time both revealed significant main effects [*F*(3,28) = 6.46, *P* = 0.002 and *F*(7,196) = 139.88, *P* < 0.0001, respectively]. The interaction effect revealed a significant difference [*F*(21,196) = 4.09, *P* < 0.0001], showing the differing effects of different microbes on growth. The GF and *E. rectale* groups were significantly higher than the *L. plantarum* and *C. coccoides* groups (*P* < 0.05) at the 2nd and 7th weeks under both sedentary and training conditions. No significant difference in body composition among the groups in terms of the heart, liver, muscle, spleen, lung, and kidney was observed. The only significant differences in this aspect were observed in the cecum [*F*(3,32) = 11.274, *P* < 0.0001] and EPF [*F*(3,32) = 3.4, *P* = 0.038] tissues (Table 3), with the *L. plantarum* group exhibiting significantly lower values than the GF and *E. rectale* groups in cecum and EPF tissue after completing an additional *post hoc* test.

**TABLE 3:** The body compositions in indicated gnotobiotic animals.

	**Germ Free**	***E. rectale***	***L. plantarum***	***C. coccoides***
Heart	0.13 ± 0.02	0.13 ± 0.02	0.11 ± 0.01	0.13 ± 0.03
Liver	1.15 ± 0.63	1.07 ± 0.06	1.01 ± 0.10	1.12 ± 0.04
Muscle	0.26 ± 0.03	0.26 ± 0.02	0.25 ± 0.01	0.27 ± 0.02
Spleen	0.07 ± 0.03	0.07 ± 0.03	0.06 ± 0.02	0.06 ± 0.01
Lung	0.16 ± 0.02	0.16 ± 0.02	0.15 ± 0.02	0.15 ± 0.02
Kidney	0.33 ± 0.03	0.33 ± 0.04	0.32 ± 0.03	0.32 ± 0.04
Cecum	3.73 ± 0.82^b^	3.76 ± 0.80^b^	2.57 ± 0.42^a^	2.07 ± 0.21^a^
EPF	0.32 ± 0.04^b^	0.31 ± 0.02^b^	0.25 ± 0.06^a^	0.28 ± 0.03^ab^

				(Gram)

*Values in the same row with different superscript letters (a,b) differ significantly, *p* < 0.05, by one-way ANOVA. EPF, epididymal fat pad.*

### Histopathological Observations

**FIGURE 9:**
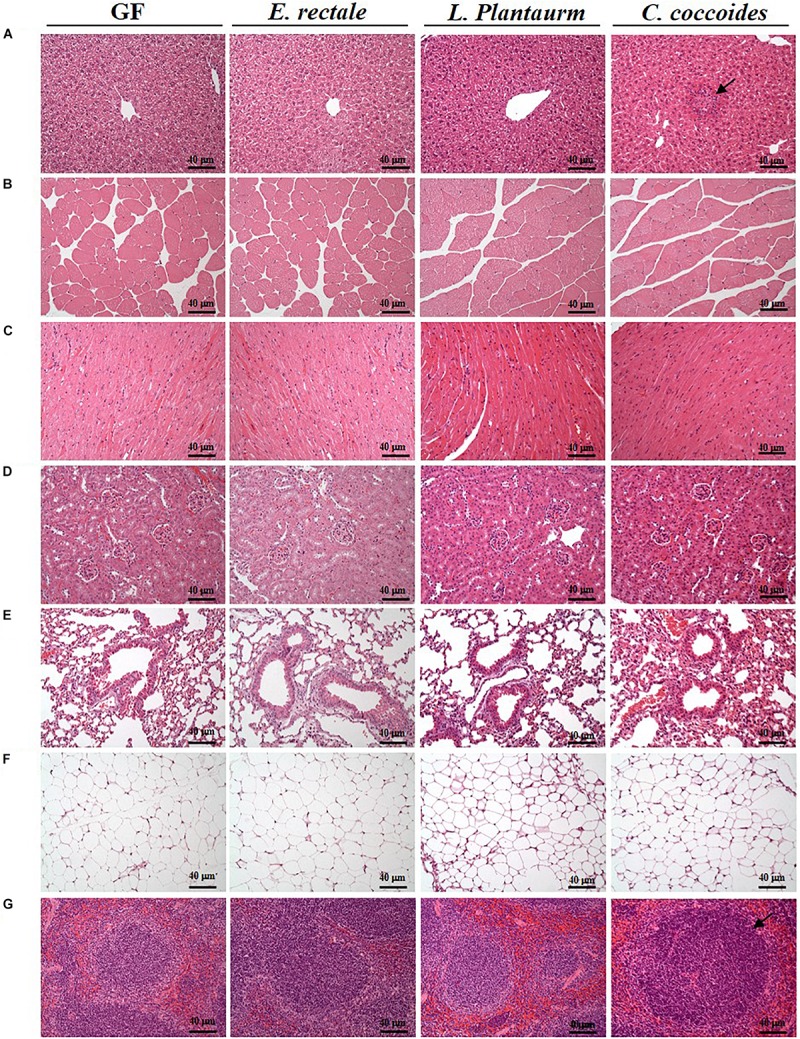
Effect of indicated gnotobiotic groups on histomorphologic features of the liver **(A)**, muscle **(B)**, heart **(C)**, kidney **(D)**, lung **(E)**, EFP **(F)**, and Spleen tissue **(G)**. Specimens were photographed under a light microscope. (H&E stain, magnification: 200×; bar, 40 μm). The arrows mean the necrosis and hypertrophy in the liver and spleen, respectively. EFP, Epididymal fat pad.

**FIGURE 10:**
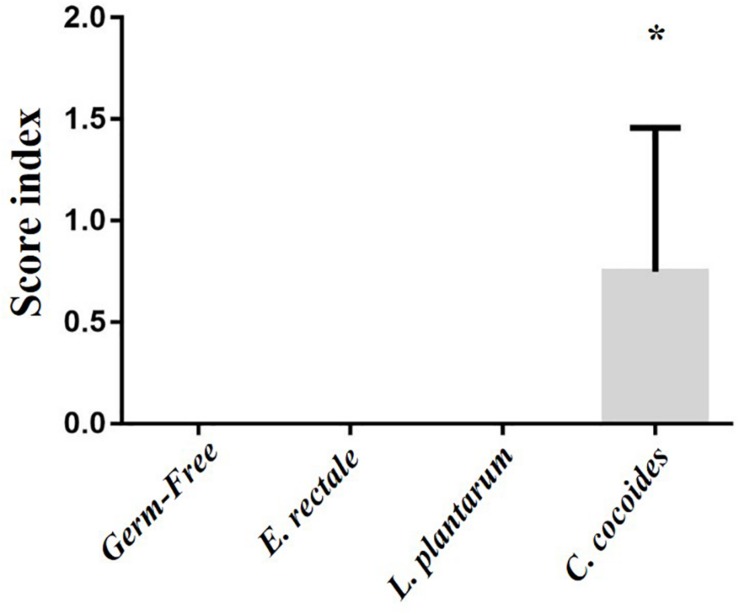
Effect of indicated gnotobiotic groups on pathological evaluation. Data are mean ± SEM (*n* = 6–8/group) and ^∗^*P* < 0.05, mean the significant different as compared with GF, *E. rectale*, and *L. plantarum* groups.

As shown in Figure 9, a histological examination of the main organs, including the liver, spleen, kidney, muscle, heart, EFP, and lungs, in the indicated gnotobiotic groups were observed at the end of study, demonstrating the representative photomicrographs of the organs from the indicated groups. HE staining of liver samples showed normal hepatic architectures such as hepatocytes, bile duct, and sinusoid in the GF, *E. rectale* and *L. plantarum* groups. However, only minimal levels of focal hepatocyte necrosis were observed in the *C. coccoides* group (Figure 9A). Interestingly, significantly smaller amounts of glycogen were found in the gnotobiotic groups than the GF group. This morphologic change was consistent with the results of hepatic glycogen quantification analysis. In the spleen, the zones of white pulp were more enlarged in the *C. coccoides* group than in other groups (Figure 9G). Histological analysis of the kidney showed a normal architecture with glomerulus, tubules, and interstitial tissue. Muscles hypertrophy and hyperplasia were not observed in the heart cardiomyocytes and rhabdomyocytes of gastrocnemius muscle. The lung sections were normal in appearance, with normal bronchioles and alveoli. In terms of fat tissue, relative smaller vesicle size of lipids on EFP were found in the *L. Plantarum* group compared with the GF and *E. rectale* groups. The pathological scoring standards for non-neoplastic lesions was based on previous published report (Shackelford et al., 2002) and the minimal to mild hepatic necrosis lesion in liver was significantly higher than other groups (Figure 10).

## Discussion

The present study established a gnotobiotic animal model with indicated microbes, including *E. rectale*, *C. coccoides*, and *L. plantarum* TWK10, in order to verify the physiological adaptions that result from exercise. *E. rectale* demonstrated a positive effect on exercise performance both with and without exercise training intervention. This positive effect may relate to the improved bioavailability of energy. After the acute exercise challenges, the fatigue-associated indexes were significantly improved in the *E. rectale* and *C. coccoides* groups, consistent with their exercise capacity. In the metabolic phenotype analysis, the behavior of physical activity was also more upregulated in the three gnotobiotic groups than in the GF group. In addition, the BMR of the *L. plantarum* TWK10 and *C. coccoides* groups was significantly higher than that of the other groups. This finding may be attributed to both growth and body composition. In the gnotobiotic model, *E. rectale* showed potential benefits on exercise physiology and energy availability.

Sports nutrition, an emerging area of focus in the study and practice of nutrition and diet, offers the potential to improve the health of both athletes and the general population. As all nutrients must be ingested, digested, and absorbed by the GI tract, intestinal microbes may play important roles not only in assisting nutrient digestion and absorption but also in homeostatic maintenance of host immunity, metabolism, and the gut barrier as a symbiotic system (Van de Guchte et al., 2018). Many previous studies have examined the contribution of exercise to positive changes in microbiota composition. In previous study, the exercise intervention was found to reduce the quantity of Bacteroidetes and increase the quantity of Firmicutes, with related changes highly correlated with VO_2_ max (Durk et al., 2018). Besides, it also demonstrated the greater abundance of select Firmicutes species and lower *Bacteroides*/*Prevotella* spp. in exercised mice compared with sedentary counterparts (Lambert et al., 2015). Prior studies have endemically isolated *L. plantarum* TWK10, belonging to the phylum Firmicutes, from Taiwanese pickled vegetables and elucidated its potential efficacies on exercise physiology (Chen et al., 2016; Huang et al., 2018a). In addition, the detailed taxonomy of microbiota have also been reported in an exercise intervention, which further found a significant increase in *Clostridium* spp. with regular exercise in similar proportions and abundances in both mice and humans (Nguyen et al., 2015; Campbell et al., 2016). *E. rectale*, a butyrogenic microbe, has been mainly reported with regard to the physiology effects on SCFAs such as exergy metabolism, intestinal integrity, anti-oxidation, gene regulation, and insulin resistance (Tan et al., 2014). Therefore, this study applied these three microbes in a gnotobiotic model to investigate their direct effects on physiological adaption, capacity, and metabolism.

Exercise interventions have been shown to significantly increase the populations of *Lactobacillus*, *Bifidobacterium*, and *E. rectale* in animal studies (Queipo-Ortuño et al., 2013). In addition, the association between cardiorespiratory fitness (VO_2_ max) and *EreC* (*Eubacterium rectale*–*Clostridium coccoides*) appears to be mediated by body fatness and physical activity, which alters the gut microbiota composition and affects microbiota diversity in a manner similar to nutritional status (Yang et al., 2017). This has elucidated the relationship between exercise and increased levels of *E. rectale*, a microbe that has been linked to the production of SCFAs and multiple related physiological benefits (Koh et al., 2016). This study found that the *E. rectale* in the gnotobiotic model significantly increased exercise capacity, both with and without aerobic training, in comparison with the other groups with main (microbe) and interaction (microbe × time) effects. Remarkably, *L. plantarum* TWK10 did not significantly improve exercise capacity, which was inconsistent with previous studies (Chen et al., 2016; Huang et al., 2018a). A possible explanation is that *L. plantarum* could not exert its bioactivities with the gnotobiotic model and that diversity of microbiota for mutual interaction may be critical to improving exercise capacity and physiological adaption. Combinations of probiotics and prebiotics may be further studied using the gnotobiotic model to identify and describe additional synergistic effects.

The indexes that are associated with exercise-related physiological adaption, including lactate, ammonia, LDH, CK, and glucose, have been generally applied in assessments of physiological status (Huang et al., 2018b). The GF mice demonstrated extremely weak capacities for exercise and adaptation, possible due to the anti-oxidant ability provided by *Bacteroides fragilis* microbiota (Hsu et al., 2015). This negative impact on exercise capacity and training adaption was observed in this study (Figure 2). We proposed that the microbiota may be involved not only in the anti-oxidative system but also in critical systems providing and regulating energy. The results of this study firstly revealed the GF-related exercise physiological indexes mentioned above, which were obviously higher in GF lactate and ammonia levels after the exercise challenge, with variabilities attributable to species and microbiota status (Hsu et al., 2018). The *E. rectale* group significantly ameliorated the increase of exercise-influenced lactate and ammonia indexes, as shown in Figures 4A,B, and the glucose was significantly elevated to meet the energy demand of exercise (Figure 6A). Furthermore, the SCFA acted as signaling molecules, helping regulate the hepatic lipid and glucose homeostasis in an adenosine monophosphate-activated protein kinase (5′ AMP-activated protein kinase) dependent manner involving peroxisome proliferator-activated receptor-γ (PPAR-γ) regulated effects on gluconeogenesis and lipogenesis and the butyrate could activate the gluconeogenic gene for a homeostatic signal in the hepatic portal system (Morrison and Preston, 2016). Moreover, a previous study elucidated that SCFA excretions could also upregulate the mRNA expression of PPAR-γ, glucose transporter-4 (GLUT4), phosphoinositide 3-kinase (PI3K), and phosphorylated-Akt (p-Akt) and improve glucose uptake by inducing GLUT4 translocation through PI3K/Akt signaling pathways (Zhu et al., 2016). This study found elevated GLUT4 expression in muscles, possibly due to SCFA produced by *E. rectale* microbiota, which improved the glucose uptake for use in exercise (Figure 6B). In terms of glycogen content, this study revealed that the GF mice demonstrated abnormal metabolic characteristics in glycogen synthesis, and accumulation glycogen in the liver through gluconeogenesis and glycogenesis processes that were directly associated with microbiota effects (Chuang et al., 2012; Hsu et al., 2015). The glycogen of the *E. rectale* gnotobiotic animals could potentially upregulate the enzyme activities for the glycogenolysis pathway, which is consistent with the result of glucose release and utilization. Therefore, the hepatic glycogen content was significantly decreased compared to the GF group, and glycogen bioavailability could have been affected by the microbiota interactions (Figure 7).

Scheiman et al. (2019) identify the microbiota of genus *Veillonella* (*Veillonella atypica*) exerted a major pathway metabolizing lactate to propionate for higher endurance performance in elite runner by a shotgun metagenomic analysis. Therefore, we compared to our gnotobiotic mice (*E. rectale* and *C. coccoides*) with significant lactate decrease after exercise. The SCFAs, formed by microbial fermentation or supplement, could promote the fat oxidation (Canfora et al., 2017) and it could regulate significant changes in the expressions of G-protein coupled receptor (GPR) for enhancement of triglyceride hydrolysis, FFA oxidation, mitochondrial biogenesis, and anti-inflammation (Lu et al., 2016). Besides, the GLUT4 was also significantly elevated for glucose uptake (Figure 6B). The *E. rectale* gnotobiotic could increase the aerobic or endurance capacity possible by improvement of energy modulation and aerobic metabolism with lower lactate production during exercise. The *E. rectale* may exhibit the different physiological effects from *Veillonella atypica*. Remarkably, we also found the *L. plantarum* gnotobiotic didn’t exert the beneficial effects on exercise performance and physiological adaption as compared to previous normal supplement study (Chen et al., 2016) and it could need to interactively cooperate with other microbes for their physiological activity. Thus, the omics studies could be further applied to current topics to clearly realize the possible correlations of gene expression and regulation between host and microbes.

In a previous clinical trial, the prevalence of *L. plantarum*, *Bifidobacterium* genus, *Bifidobacterium longum*, *C. coccoides* and *C. leptum* were higher in a lean group than an obese group (Teixeira et al., 2013). Besides, the cecum is known to be significantly higher in weight and proportions in GF mice than in wild-type mice due to differences in anatomical and physiological characteristics (Kostic et al., 2013). This study observed that the *L. plantarum* and *C. coccoides* with gnotobiotic model significantly increased the BMR index and significantly decreased the weights of fat tissue in comparison with the other groups (Tables 2, 3). Moreover, the weight of the cecum was significantly less in comparison to other GF groups, possibly due to the significant differences in metabolic and digestive features in the *L. plantarum* and *C. coccoides* gnotobiotic model (Table 3), which gave *L. plantarum* and *C. coccoides* groups significantly less bodyweight at the end of the study. This histopathological observation may be validated for the safety and the effects on different tissues with *E. rectale* and *L. plantarum* gnotobiote.

## Conclusion

Probiotics have been widely investigated for their potential impacts on and connections to immuno-regulation, gastrointestinal functions, inflammation, and metabolic syndrome (Torres et al., 2018). However, few studies have investigated the effects of probiotics or specific microbiota activities on exercise physiology. This study used a gnotobiotic animal model to establish an assessment platform for probiotics/microbiota evaluation and attempted to identify the possible regulation mechanisms between the host and microbiota as a reference for future studies. Results indicate that the *E. rectale* microbiota has potential benefits for exercise-associated physiological adaption. Moreover, the *L. plantarum* TWK10 and *C. coccoides* microbiota may improve energy expenditure and body compositions in combination with exercise intervention. Based on the findings of this study, the synergistic effects of a single or combinational microbiota on normal microbiota or gnotobiotic model point to an interesting issue that should be addressed in future investigations of the physiological benefits of exercise. Finally, the authors of this study believe that beneficial microbiota development is an emerging field in exercise and fitness that holds significant potential for promoting health and performance.

## Ethics Statement

This study was carried out in accordance with the principles of the Basel Declaration and the recommendations of the Institutional Animal Care and Use Committee (IACUC) of National Taiwan Sport University. The protocol (IACUC-10612) was approved by the IACUC of National Taiwan Sport University.

## Author Contributions

W-CH and C-CH designed the experiments. W-CH, Y-HC, and H-LC carried out the laboratory experiments and bacteria culture. H-LC and C-CC contributed reagents, materials, and analysis platforms. W-CH, H-LC, and C-CC analyzed and illustrated the data. W-CH and C-CH interpreted the results, prepared the figures, and wrote and revised the manuscript.

## Conflict of Interest Statement

The authors declare that the research was conducted in the absence of any commercial or financial relationships that could be construed as a potential conflict of interest.
